# Copper Contamination Impairs Herbivore Initiation of Seaweed Inducible Defenses and Decreases Their Effectiveness

**DOI:** 10.1371/journal.pone.0135395

**Published:** 2015-08-14

**Authors:** Alexandria M. Warneke, Jeremy D. Long

**Affiliations:** 1 Department of Biology, San Diego State University, San Diego, California, United States of America; Universitat Bremen, GERMANY

## Abstract

Seaweed-herbivore interactions are often mediated by environmental conditions, yet the roles of emerging anthropogenic stressors on these interactions are poorly understood. For example, chemical contaminants have unknown consequences on seaweed inducible resistance and herbivore response to these defenses despite known deleterious effects of contaminants on animal inducible defenses. Here, we investigated the effect of copper contamination on the interactions between a snail herbivore and a brown seaweed that displays inducible resistance to grazing. We examined seaweed inducible resistance and its effectiveness for organisms exposed to copper at two time points, either during induction or after herbivores had already induced seaweed defenses. Under ambient conditions, non-grazed tissues were more palatable than grazed tissues. However, copper additions negated the preference for non-grazed tissues regardless of the timing of copper exposure, suggesting that copper decreased both how herbivores initiated these inducible defenses and their subsequent effectiveness. Copper decreased stimulation of defenses, at least in part, by suppressing snail grazing pressure—the cue that turns inducible defenses on. Copper decreased effectiveness of defenses by preventing snails from preferentially consuming non-grazed seaweed. Thus, contaminants can potentially stress communities by changing seaweed-herbivore interactions mediated via inducible defenses. Given the ubiquity of seaweed inducible resistance and their potential influence on herbivores, we hypothesize that copper contamination may change the impact of these resistant traits on herbivores.

## Introduction

Environmental stressors, occurring together or in sequence, may create surprising, interactive effects on communities [1–2]. Unfortunately, our ability to predict these ecological surprises [2] is limited due to a historic bias for studies examining the effects of individual stressors. For example, the grazing history of seaweed individuals, especially brown algae, often influences their susceptibility to future herbivory via induced resistance [3–5]. Likewise, abiotic stressors such as depleted and enriched nutrient levels [6–8], desiccation [9–10], UV irradiance [7,10], temperature [11], and light availability [12–13] can directly and indirectly influence herbivory on seaweeds. However, the outcomes of induced seaweed resistance are uncertain given that most studies tested for their presence in the absence of abiotic stress. This is particularly true for emergent stressors that have unknown impacts on inducible defenses but might commonly influence their effectiveness via information disruption [14–15].

Chemical contaminants are emergent stressors to aquatic habitats that may influence plant-herbivore interactions via changes in inducible resistance. Although studies examining impacts of contaminants on plant resistance are rare, several studies demonstrated negative impacts of contaminants on induced defenses of animals. For example, contaminants reduced the expression of defensive structures of *Daphnia* [16–19] and prevented expression of defensive behaviors in juvenile salmon and trout [20–21]. Importantly, contaminant-mediated weakening of responses to alarm cues increased salmon susceptibility to predation [21]. Similar effects of contaminants on inducible resistance of aquatic plants and seaweeds might occur via at least three pathways. First, contaminants can decrease grazing rates [22–24] and subsequently weaken the cue that initiates these defenses. Second, contaminants acting as stressors can directly affect plant traits [6,9,10], some of which may be related to these defenses. Third, contaminants may interfere with chemoreception and thereby prevent herbivores from recognizing inducible defenses of macrophytes [14–15].

In the only known test of the effects of chemical contaminants on inducible seaweed defenses, copper exposure did not alter phlorotannin production in the seaweed *Ascophyllum nodosum* [25]. Previous studies by these authors demonstrated that phlorotannins, a class of polyphenolic compounds that have been hypothesized to provide a defensive function (but see [26–27]) became elevated after herbivore attack as part of an inducible response [3,28]. However, Toth and Pavia [25] only examined the direct effect of copper on phlorotannin production as opposed to the indirect effect of copper on phlorotannins via changes in herbivore behavior. Given that many of the proposed pathways by which contaminants affect inducible resistance are mediated by changes in herbivore behavior (see above), we need studies that test for these effects.

While copper plays a key role in the metabolism of aquatic invertebrates [29], human activities can elevate copper within coastal embayments to levels that negatively affect marine organisms [30,31]. Furthermore, contaminant effects on plant defenses might be particularly common in brown seaweed (Phaeophyceae) because they 1) commonly display inducible resistance [5] and 2) can hyper-accumulate heavy metals [32,33]. Using a model seaweed that displays inducible resistance to herbivory, we investigated the influence of copper contamination on the herbivores ability to initiate seaweed inducible defenses and the subsequent effectiveness of these defenses.

## Materials and Methods

### Study Organisms, Copper Levels, and Overall Design

To examine the influence of chemical contamination on interactions mediated by seaweed inducible defenses, we selected a model-system consisting of the fucoid seaweed (*Silvetia compressa*) that reliably becomes less palatable after grazing by snails (*Tegula funebralis*) in uncontaminated scenarios [Jones and Long, *unpublished data*]. Our experiments exposed organisms to two copper levels: Ambient and Elevated. Seawater used to create these treatments was obtained from the flow-through seawater system at the Scripps Institution of Oceanography pier. All experimental seawater was passed through a 0.45-micron filter to remove particulates that could decrease copper bioavailability via precipitation. Copper levels in this seawater system are typically low (<2.0 μg/L)[34]. Thus, we refer to the low level as “Ambient.” Our “Elevated” treatments increased copper above these background levels by 50 μg/L.

We selected our Elevated copper level to represent maximum levels that are currently encountered in marine environments. Similar or higher levels of copper have been observed in coastal environments in Texas (50 μg/L)[35], Chesapeake Bay (70 μg/L)[36], and England (>100 μg/L)[37]. Furthermore, there is evidence that another fucoid seaweed with well-known inducible resistance (*Fucus vesiculosus*) can encounter such copper levels and will accumulate copper in its tissues at these sites [37].

We created copper solutions by diluting a stock solution (1 mg/L copper chloride, CuCl_2_) with filtered seawater collected from the pier at Scripps Institution of Oceanography. Stock solutions were created with analytical grade copper chloride (CuCl_2,_ Sigma-Aldrich Co.). We verified the concentration of these solutions through an independent laboratory (0.973 mg/L copper chloride, CuCl_2_, Enviromatrix Analytical Inc., San Diego, CA). Stock solutions were stored at 12°C to minimize reduction of chemical ions in solution. All containers and materials used for chemical exposure were acid washed using a 10% Nitric Acid solution and thoroughly rinsed with deionized water prior to all experiments [38]. Experimental protocols involving copper waste, disposal, and organism handling were strictly adhered to and approved by San Diego State University-Department of Environmental Health and Safety.

We collected all organisms from the high intertidal zone of La Jolla, California, USA (32°48’39” N, 117°16’10” W; CA-DFW Permit # SC-12419) where these species co-occur at high densities and transported them to San Diego State University’s Coastal and Marine Institute Laboratory (CMIL). Prior to experiments, we maintained organisms for 72 h in an outdoor, flow-through seawater system with tanks that drained and flooded to mimic semi-diurnal tidal conditions. Snails were fed *Silvetia* ad libitum and then starved for 24 h leading up to experimentation to ensure even satiation across all treatments.

Seaweed induction experiments followed the standard approach for assessing inducible defenses, whereby defenses were elicited during an induction phase and the effectiveness of these defenses were measured during a separate bioassay phase [3–4, 39–40]. The induction phase lasted for 14 d. Because copper effects may depend upon the timing of exposure relative to the timing of induction, we examined the consequences of copper pollution during both the induction phase and bioassay phase.

### Effects of contamination during induction

To investigate the influence of copper on herbivore initiation of inducible defenses in *Silvetia*, we conducted the induction phase under Ambient or Elevated copper levels. During this phase, *Silvetia compressa* was grown individually in the presence or absence of ecologically realistic densities of *Tegula funebralis* (8 *Tegula* per 20 ± 0.2 g *Silvetia* **)** for 14 d. This ratio (Number of *Tegula* per *Silvetia* biomass) represents the maximum that we have observed at our field sites in San Diego. Individual seaweeds were placed in replicate containers (1 L volume; 190 x 165 x 91 mm, n = 20). Plastic window screen mesh (6 mm openings) covered openings on all containers to prevent snail escape.

Replicate containers were maintained in two large, separate recirculating seawater systems (either Ambient or Elevated, 19 ± 1.0°C), Replicates were randomly interspersed to minimize any potential positional effects. To create circulation, water was pumped from a large sump tank (40 L/min flow rate), into a large tank holding the replicate containers, and then drained into the sump. An automatic timer set to mimic times of tidal flux, regulated when the pump was active (organisms exposed to high tide) and inactive (organisms exposed to low tide). In these systems, the total volume of water (340 L) was replenished daily for both systems to minimize nutrient loss and waste accumulation (both treatments) and copper loss (Elevated treatment only). Recirculating seawater systems were illuminated by fluorescent tubes (85.6 ± 5 μmol photons • m^-2^ • s^-1^ (PAR); Philips Natural Light 40W) placed on a 12:12 hr light/dark cycle.

Following the 14 d induction phase, *Silvetia* were removed from Ambient and Elevated treatments, re-weighed to quantify herbivore grazing changes during the Induction phase, and offered to another set of *Tegula* (3 *Tegula* per 3 ± 0.1 g *Silvetia*) in a paired choice bioassay for 5 d (Grazed vs. Non-grazed). Similar to other studies, our choice bioassays were conducted to identify feeding preferences of herbivores. We used higher ratios of snails to seaweed biomass to maximize the feeding signal that would allow us to detect snail feeding preferences. Grazed and Non-grazed pieces from the Ambient and Elevated Induction treatments, were marked with colored rubber bands and paired. We measured the initial wet mass of each seaweed (T_i_) and placed pairings in an uncontaminated bioassay. Bioassays ran for 5 d in separate, aerated containers randomly interspersed within a temperature- and light-controlled growth chamber (19 ± 1.0°C, 85.6 ± 5 μmol photons • m^-2^ • s^-1^ (PAR); Philips Natural Light 40W, 12:12 hr light/dark cycle). We replaced the water in these containers daily to maintain nutrient levels (*sensu* [28]). After 5 d of grazing, we measured the final wet mass of each seaweed (T_f_). The snail-free controls were interspersed with snail treatments.

### Effects of contamination after induction

To examine the influence of copper contamination occurring after induction on the effectiveness of these defenses, we repeated the above experiments with two major changes. First, the induction phase was only conducted in Ambient conditions. Second, organisms were exposed to Ambient or Elevated copper levels during the bioassay phase.

*Silvetia* was collected at the end of a new 14 d uncontaminated induction phase and subsequently offered to another set of *Tegula* (3 *Tegula* per 3 ± 0.1 g *Silvetia* **)** in a choice assay. During this 5 d bioassay phase, Grazed and Non-grazed tissues were offered as pairs to *T*. *funebralis* in Ambient or Elevated copper exposures (50 μg/L). Under Ambient conditions, *Silvetia* displaying induced resistance after growing in the presence of snails during the induction phase should be less palatable than seaweeds that grew in the absence of snails. To prevent the release of copper into our seawater system effluent, all bioassays ran for 5 d in individual replicate containers within a temperature- and light-controlled growth chamber (19 ± 1.0°C, 85.6 ± 5 μmol photons • m^-2^ • s^-1^ (PAR); Philips Natural Light 40W, 12:12 hr light/dark cycle). We replaced the water in these containers daily to maintain nutrient and copper levels (*sensu* [28]).

To determine if induced and non-induced seaweeds accumulated similar levels of copper during the bioassay phase, we measured copper levels with a separate set of induced and non-induced seaweeds exposed to elevated copper for five days after the induction phase (n = 8). As a reference, we also measured copper levels in seaweeds taken directly from our field site. Prior to copper analysis, tissues were prepared using a modified acid digestion protocol [41]. Specifically, we digested 1.5 g of dry-blotted wet tissue in glass tubes with 5 mL of HNO_3_ for 14 h at 120–130°C. After this, we added 1mL hydrogen peroxide and diluted samples to a total volume of 50 mL with double-distilled water. Samples were further diluted to 2% acid concentrations for trace analysis. Copper concentrations were analyzed using inductively coupled plasma mass spectrometry (ICP-MS, Agilent Technologies 7900 with a CETAC Technologies ASX-520 autosampler) and adjusted to amount of copper per dry mass by calculating a dry/wet ratio from a separate set of dried tissues.

### Decoupling organismal exposure and influence of exposure duration

Because copper reduced the effectiveness of seaweed inducible defenses, we conducted experiments to identify the mechanisms underlying this change. In particular, we studied the role of exposure duration to contaminants and the impact of only exposing snails to contaminants. We grew *Silvetia* with and without herbivores for 14 d during an uncontaminated induction phase (8 *Tegula* per 20 ± 0.2 g *Silvetia)*. Grazed and Non-grazed seaweeds from this induction phase were paired during choice bioassays (3 *Tegula* per 3 ± 0.1 g *Silvetia* **)** conducted under four different copper exposures (n = 20). As before, the Ambient treatment consisted of filtered seawater without copper additions. We expected herbivores to prefer Non-grazed seaweeds in this Ambient treatment. The three remaining treatments (Acute, Chronic, Chronically exposed snails) exposed at least one organism to copper. The Acute treatment exposed both snails and seaweed to elevated copper (50 μg/L) for one day, and then measured snail feeding preference for Grazed and Non-grazed tissues in uncontaminated conditions for the duration (4 d) of the bioassay phase. In contrast, the Chronic treatment exposed both snails and seaweeds to copper for the entire duration (5 d) of the bioassay phase.

Our fourth treatment, Chronically exposed snails, allowed us to examine the influence of chronic exposure of snails independent of effects on seaweeds. We created contaminated stocks of snails in a separate tank containing *Silvetia* and seawater with 50 μg/L CuCl_2_. Copper exposures of these snail stocks were staggered so that each snail set received the same amount of copper exposure per day as those in the other treatments. For the duration of the 5 d bioassay phase, contaminated snails (3 *Tegula* per day) were then placed into uncontaminated bioassay treatments containing paired Grazed and Non-grazed *Silvetia*. Snails were removed from replicate containers during daily water changes and new sets of snails were rotated in from the contaminated stock to maintain consistent levels of herbivore contamination. Snails were not re-used in the bioassay on multiple days.

### Statistical Analysis

Grazing rates were corrected for autogenic seaweed growth in an equivalent number of snail-free controls using the formula T_i_(U_f_/U_i_)-T_f_ where U_i_ and U_f_ represented the initial and final masses of Non-grazed tissue from controls, respectively [42]. Correcting for autogenic seaweed growth allowed us to explore changes in feeding preference unrelated to differences in seaweed growth. We first examined the influence of copper on snail feeding rates by comparing corrected consumption rates in Ambient versus Elevated treatments with a two-tailed, two-sample t-test. We used repeated measures analysis of variance (RM-ANOVA) to examine the effect of copper pollution on feeding preference for Grazed and Non-grazed tissues during each of our three major experiments. Prior to statistical analysis, adjusted masses of Grazed and Non-grazed *Silvetia* tissues (T_i_(U_f_/U_i_)-T_f_) were first tested and confirmed for meeting the assumptions of a normal distribution and homogeneity of variances. Repeated measures analysis was necessary due to lack of independence between tissues types within each replicate. Snails were offered tissues in pairs. This statistical method has been increasingly utilized when choice assays are used in more complex designs (i.e. those with multiple factors [40,43]). In these repeated measures ANOVAs, the between-subjects factor was Copper contamination (Ambient, Elevated) and the within-subject factor was Induction status (Grazed, Non-grazed). To reveal which combination of copper exposure treatments caused significant interactions, paired t-tests were performed as post-hoc comparisons [40]. We confirmed that all paired t-tests met the normality assumption using the Kolmogorov-Smirnov test. Lastly, we tested the difference in dry tissue copper levels (Grazed, Non-grazed, Field collected) using a one-way analysis of variance (ANOVA). Concentration values were square root transformed to meet the assumption of equal variances.

## Results

**Fig 1:**
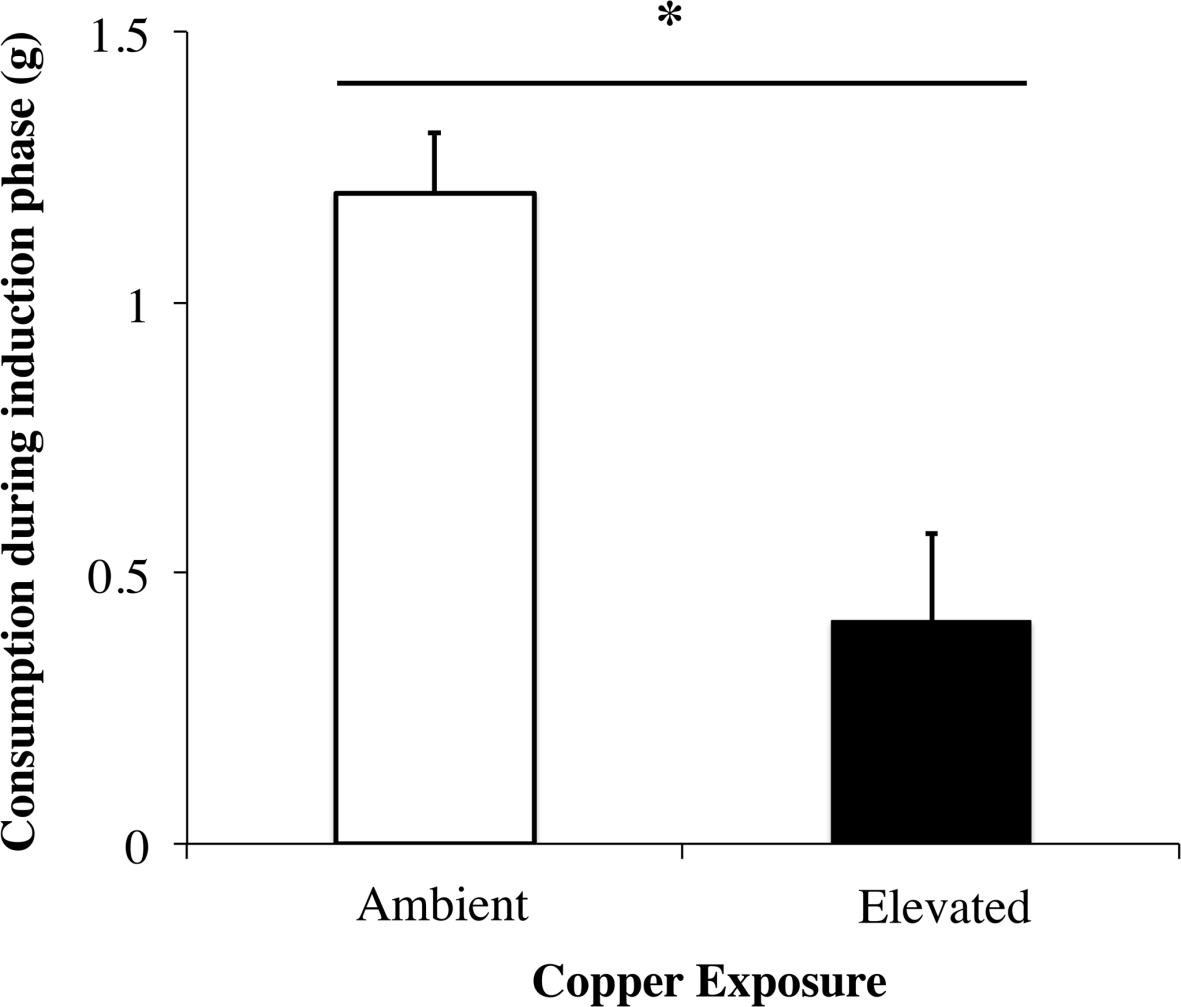
Snail Consumption Under Copper Exposure. Mean consumption (±SE) of *Silvetia compressa* by *Tegula funebralis* under Ambient and Elevated copper exposures. For Elevated treatments, both herbivores and seaweeds were exposed to 50 μg/L Cu for 14 d. Asterisk (*) designates statistical difference of two-sample t-test (p<0.05).

**Fig 2:**
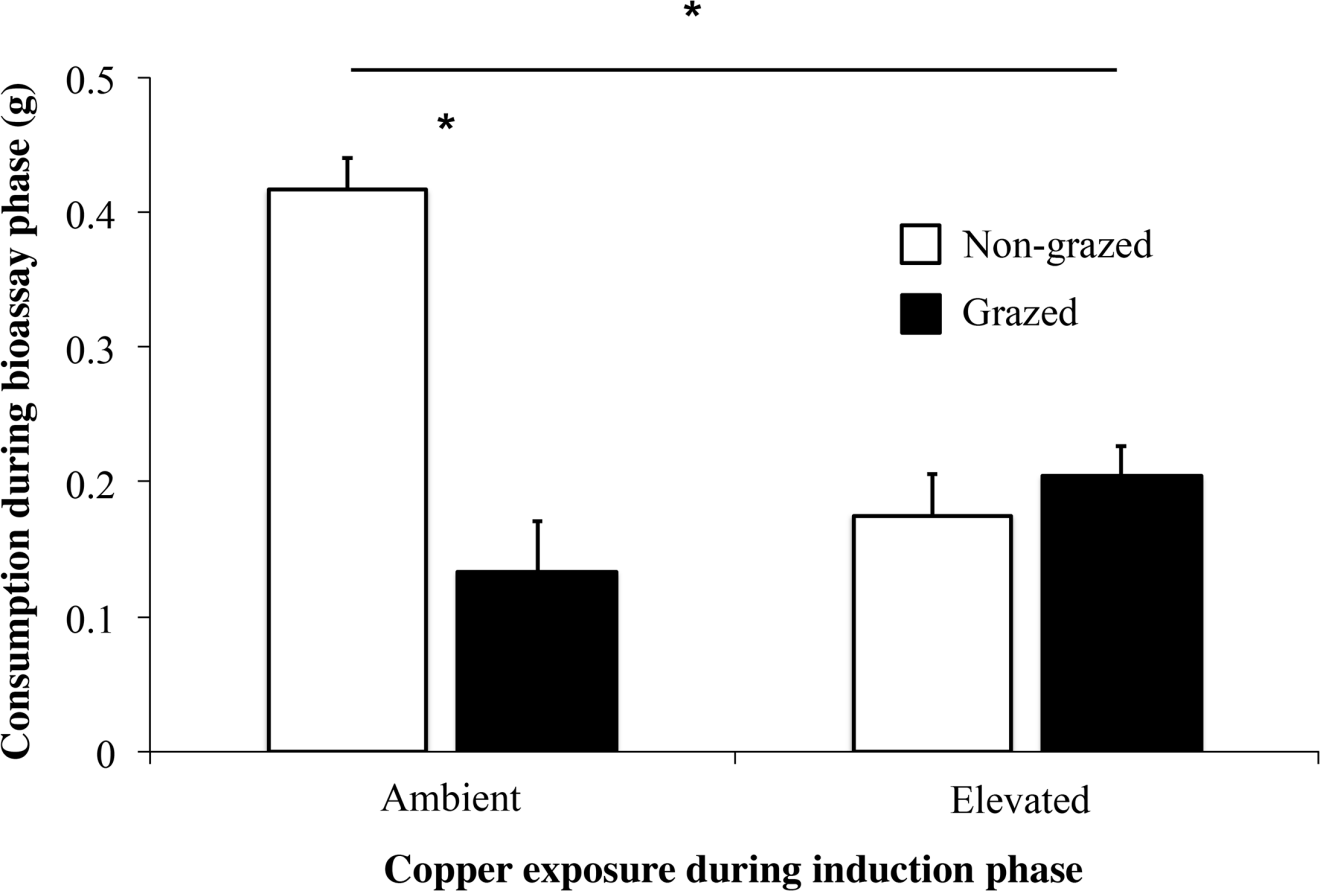
Contamination During Induction. Mean consumption (±SE) of Grazed and Non-grazed *Silvetia compressa* by *Tegula funebralis* during uncontaminated choice bioassays using seaweeds that were previously induced with snails under Ambient and Elevated copper exposures. Grazed seaweeds are denoted by black bars and Non-grazed seaweeds by white bars. Asterisks (*) above horizontal bars designate statistical difference of repeated measures test (p<0.05). Asterisks (*) found above paired Ambient bars designate statistical difference between Grazed and Non-grazed tissues (p<0.05).

Elevated copper concentration decreased snail feeding by approximately 66% (Fig 1, t_38_ = 4.01, p<0.001). Snail feeding preferences during the bioassay phase depended upon previous exposure of snails and seaweeds to copper during the induction phase (Copper contamination x Induction status interaction F_1,38_ = 34.0; See Fig 2, Table 1). Specifically, snails preferred Non-grazed seaweeds exposed to ambient copper levels during the induction phase (t_19_ = 7.13, p<0.001). In contrast, snails did not distinguish between Grazed and Non-grazed seaweeds when previous grazing occurred with elevated copper levels (Elevated treatment, t_19_ = 0.80, p = 0.432). Thus, elevated copper during the induction phase influenced herbivore initiation of seaweed inducible defenses.

**Table 1:** Repeated measures ANOVA for mean consumption of seaweeds (Grazed, Non-grazed) contaminated in the induction phase (Elevated, Ambient) and placed in a subsequent uncontaminated paired choice assay.

Source of Variation	SS	df	MS	F	*P*
Between subjects					
Contamination status	0.145	1	0.145	7.295	**0.010**
Error	0.756	38	0.02		
Within subjects					
Grazing status	0.324	1	0.324	22.556	**0.000**
Contamination status	0.487	1	0.487	33.948	**0.000**
•Grazing status					
Error	0.545	38	0.014		

This analysis had a between-subjects factor (Contamination status) and a within-subject factor (Grazed status). Bold values indicate significance (p<0.05).

**Fig 3:**
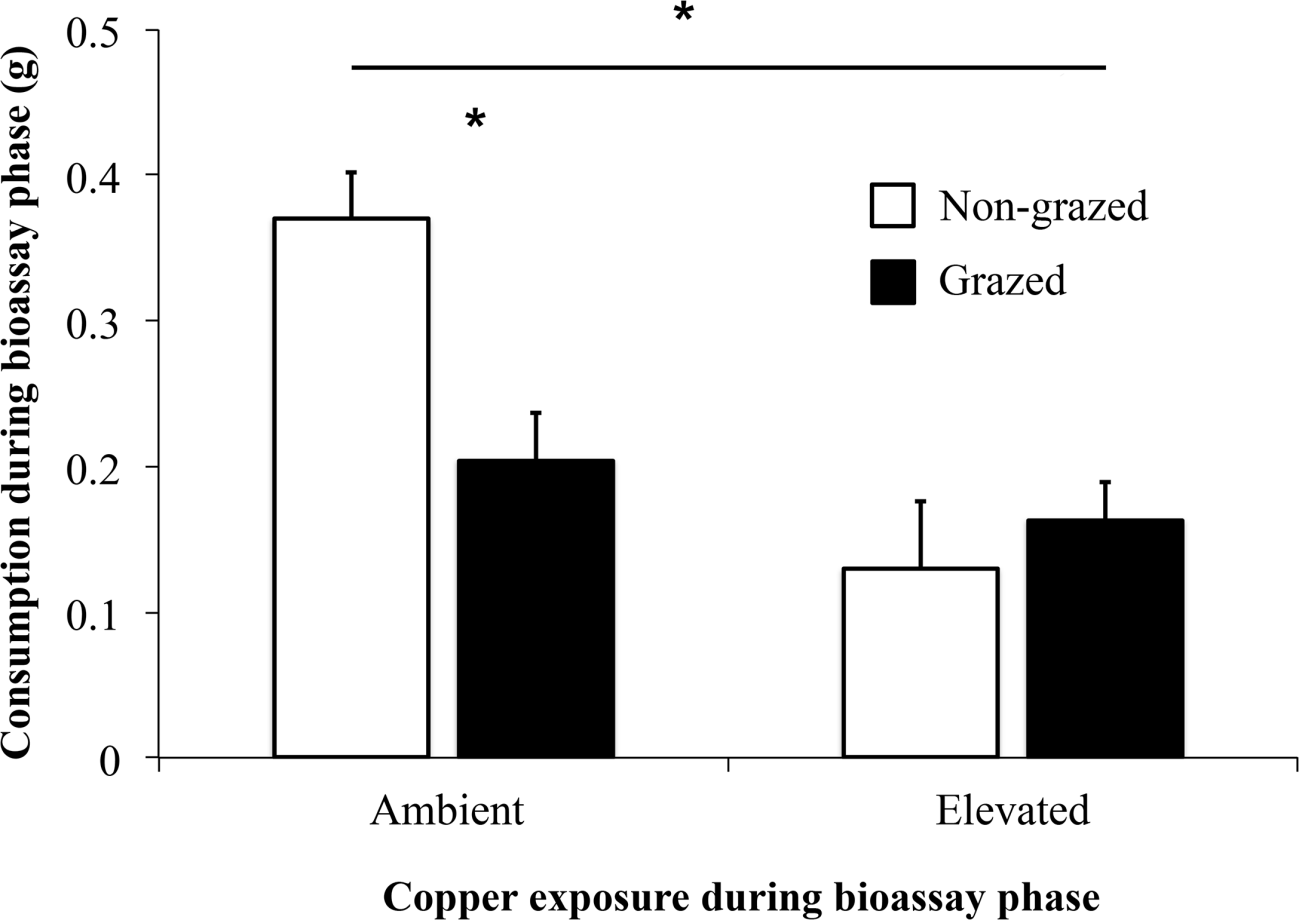
Contamination after Induction. Mean consumption (±SE) of Grazed and Non-grazed *Silvetia compressa* by *Tegula funebralis* in choice bioassays under Ambient and Elevated copper exposures. Induction occurred in the absence of elevated copper levels. Grazed seaweeds are denoted by black bars and Non-grazed seaweeds by white. Asterisks (*) above horizontal bars designate statistical difference of repeated measures test (p<0.05). Asterisks (*) found above paired Ambient bars designate statistical difference between Grazed and Non-grazed tissues (p<0.05).

Copper exposure after induction (i.e. during the bioassay phase) interacted with grazing history of the seaweed (Grazed vs. Non-Grazed) to determine herbivore preference (Interaction F_1,38_ = 7.92, Fig 3, Table 2). Consistent with the previous experiment, snails preferred Non-grazed seaweeds in the Ambient copper treatment (t_19_ = 3.21, p = 0.005). In contrast, snails did not distinguish between Grazed and Non-grazed seaweeds during bioassays with Elevated copper concentrations (t_19_ = 0.70, p = 0.494).

**Table 2:** Repeated measures ANOVA for mean consumption of uncontaminated seaweeds (Grazed, Non-grazed) placed in a subsequent contaminated choice bioassay (Elevated, Ambient).

Source of Variation	SS	df	MS	F	*P*
Between subjects					
Contamination status	0.396	1	0.396	16.555	**0.000**
Error	0.910	38	0.024		
Within subjects					
Grazing status	0.086	1	0.086	3.445	0.071
Contamination status	0.199	1	0.199	7.923	**0.008**
•Grazing status					
Error	0.952	38	0.025		

This analysis had a between-subjects factor (Contamination status) and a within-subject factor (Grazed status). Bold values indicate significance (p<0.05).

**Fig 4:**
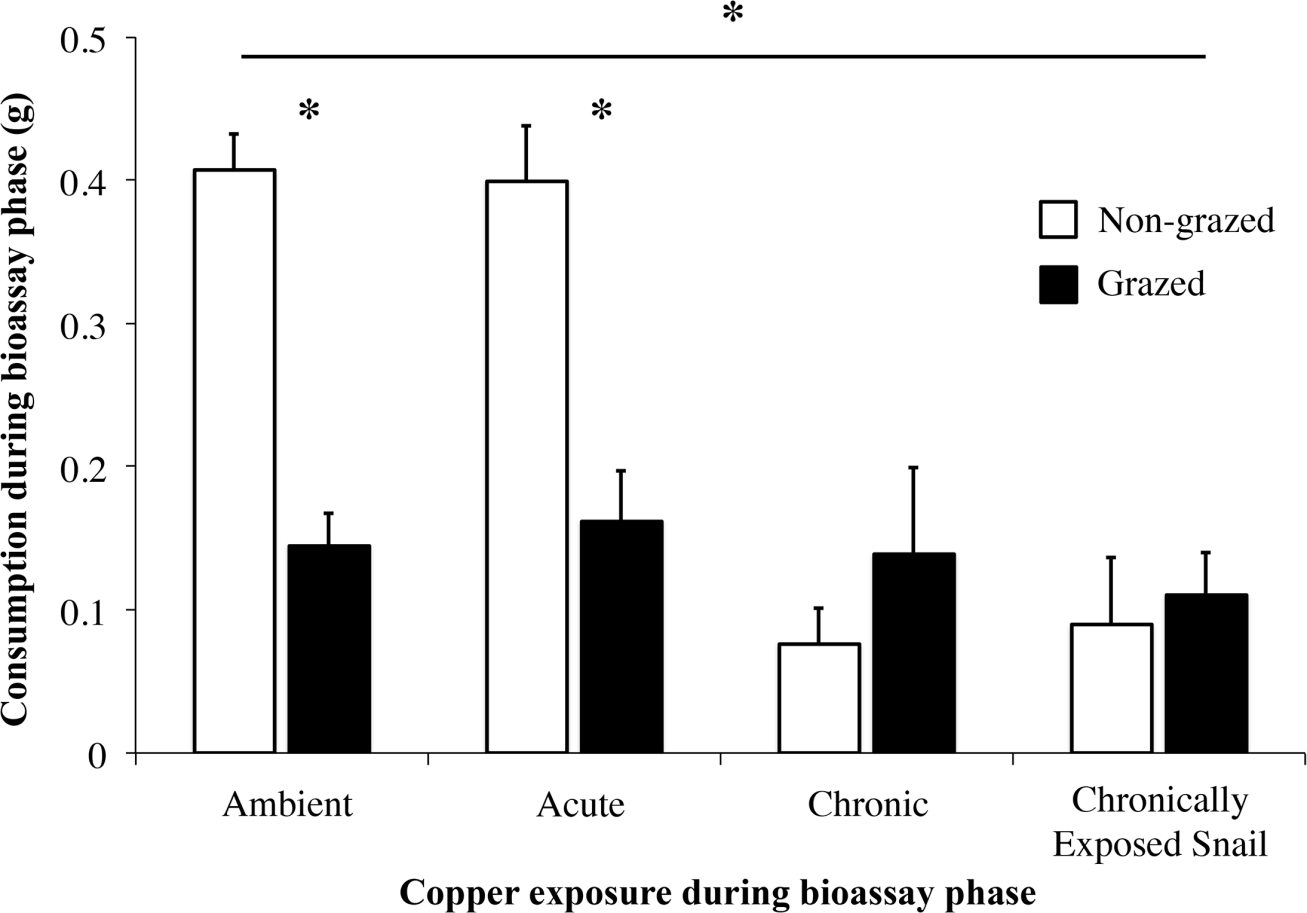
Alternation of Contamination Exposure. Mean consumption (±SE) of uncontaminated Grazed and Non-grazed *Silvetia compressa* by *Tegula funebralis* in four contaminated choice assays treatments. Exposure treatments in the bioassay phase were as follows: No exposure, Acute (exposed for only 1 d), Chronic (exposed for 5 d), and a Chronically exposed snail treatment whereby uncontaminated seaweed was grazed by chronically contaminated herbivores. Grazed seaweeds are denoted by black bars and Non-grazed seaweeds by white. Asterisks (*) above horizontal bars designate statistical difference of repeated measures test (p<0.05). Asterisks (*) found above paired Ambient bars designate statistical difference between Grazed and Non-grazed tissues (p<0.05).

In the third experiment that examined possible mechanisms by which copper influenced these species, contamination treatment interacted with grazing status to influence feeding rates (Interaction F_3,76_ = 8.75; Fig 4, Table 3). Post-hoc analyses revealed that snails preferred Non-grazed seaweeds in both the Ambient (t_19_ = 7.54, p = 0.000) and Acute exposure treatments (t_19_ = 3.91, p = 0.001). However, this preference was eliminated by exposing both snails and seaweeds (Chronic treatment, t_19_ = 0.93, p = 0.363) or just snails (Chronically exposed snails treatment, t_19_ = 0.34, p = 0.739) to copper during the bioassay phase.

**Table 3:** Repeated measures ANOVA for mean consumption of uncontaminated seaweeds (Grazed, Non-grazed) placed in a subsequent contaminated choice bioassay with four treatments (Ambient, Acute, Chronic, Chronically exposed snail only exposure).

Source of Variation	SS	df	MS	F	*P*
Between subjects					
Contamination status	1.225	3	0.408	17.476	**0.000**
Error	1.775	76	0.023		
Within subjects					
Grazing status	0.436	1	0.436	13.25	**0.000**
Contamination status	0.863	3	0.288	8.748	**0.000**
•Grazing status					
Error	2.500	76	0.033		

This analysis had a between-subjects factor (Contamination status) and a within-subject factor (Grazed status). Bold values indicate significance (p<0.05).

Seaweeds exposed to 50 ppb copper for a period of 5 d accumulated 8.973 ± 0.949 μg Cu/g seaweed dry mass and 10.517 ± 0.763 μg Cu/g seaweed dry mass (grazed and non-grazed, respectively). In contrast, field-collected seaweeds contained a significantly lower 0.584 ± 0.064 μg Cu/g seaweed dry mass (F_2,21_ = 131, p<0.001). Importantly, these are below much higher levels of accumulated tissue copper that have been observed for other fucoid seaweeds that display inducible resistance (301 μg Cu/g seaweed dry mass)[37].

## Discussion

Environmental stressors can change species interactions in surprising ways. In our study, copper contamination reduced herbivore initiation and effectiveness of inducible defenses in the brown seaweed, *Silvetia compressa*. Previous grazing reduced *Silvetia* palatability to snails in controls without added copper. However, snail preference for non-grazed tissues disappeared when copper was added during the induction of defenses or during the assessment of these induced defenses in feeding bioassays. This behavioral shift could be caused simply by exposing snails to copper for a chronic, 5 d period.

The consequences of contamination should be intimately connected to the timing of copper exposure relative to the induction status of seaweeds. If copper exposure occurs while defenses are being induced (or while induced defenses are being maintained), the strength or frequency of induced defenses should weaken because copper decreases overall grazing rates (Figs 1–4). Reduced grazing can lead to weaker inducible defenses because grazing intensity can determine defense strength [44]. Alternatively, if copper exposure occurs after defenses are induced, herbivores may consume a relatively greater proportion of defended tissues (Figs 3 and 4). Though relatively well understood in terrestrial systems [45–46], the consequences of this diet shift are difficult to predict in aquatic systems given the limited number of studies examining how inducible seaweed defenses affect herbivore performance [5]. However, previous grazing of seaweeds can suppress herbivore egg production [47] and egg hatching rates [48]. Similarly, we recently observed that *Tegula* feeding on previously grazed *Silvetia* reduces snail growth and gamete production. Thus, copper may negatively affect snail populations by preventing snails from avoiding defended tissues.

Copper may have influenced these species interactions via at least four mechanisms. First, copper contamination may have weakened herbivore stimulation of inducible plant resistance because copper suppressed grazing–the cue that elicits these responses. We found that copper decreased snail feeding by ~66%, and this reduced consumption was associated with a loss of seaweed inducible resistance. This indirect effect of copper on plant defenses may occur commonly given the well-known negative effect of copper on invertebrate feeding and survival [22–23, 49].

Second, copper may prevent herbivores from choosing non-grazed tissues versus grazed tissues because of impaired chemosensory abilities. A growing body of evidence for “information disruption” demonstrates that sub-lethal chemical contamination can act to disturb critical ecological interactions through interferences in predator perception [14]. For example, rainbow trout exposed to cadmium failed to respond to alarm chemicals [20]. More recent studies demonstrated that larval fish and foraging sharks exposed to future ocean acidification scenarios display impaired olfactory and homing abilities [43,50].

Third, copper may impair *Silvetia*’s ability to produce inducible defenses. Abiotic stressors can alter seaweed susceptibility to herbivory by modifying seaweed defensive traits [6,9,10]. In the only potential test of the effect of chemical pollution on seaweed inducible defenses, copper exposure did not induce phlorotannin production, a putative chemical defense, in *Ascophyllum nodosum* [25]. Although more research is needed, direct negative effects of heavy metals on seaweed defenses may be unlikely given the well-known ability of brown seaweeds to hyper-accumulate heavy metals without immediate detrimental effects [51]. Our study did not isolate the influence of copper on *Silvetia*’s ability to produce these defenses.

Fourth, copper accumulation in seaweeds may overwhelm herbivore preference (i.e. an elemental defense *sensu* [52]). Some terrestrial plants experience reduced herbivory after opportunistically accumulating metals [24, 52–53]. In support of this hypothesis, we found that *Silvetia* hyper-accumulated copper from contaminated waters relative to field-collected individuals. Like *Silvetia*, other brown seaweeds are well-known for their ability to accumulate high levels of copper. Copper-exposed *Ascophyllum nodosum* contained 38.9 μg Cu/g [25] and field-collected *Fucus vesiculosus* contained 301 μg Cu/g [37]–levels much higher than were accumulated by *Silvetia* in our study.

Environmental stressors can create surprising ecological effects on communities [1–2]. For example, there is a growing appreciation that contaminants can release primary producers from top-down control via direct negative effects on herbivores [49,54]. Here, we argue that contaminants may indirectly affect seaweed communities by decreasing herbivore initiation and effectiveness of seaweed inducible defenses. The ultimate consequences of contamination appear to rest heavily on the timing of exposure relative to the timing of induction. If contamination occurs during defense induction, herbivore grazing will be suppressed resulting in lower expression of seaweed defenses. The increased susceptibility of these seaweeds may be countered by an overall reduction in feeding rates of exposed herbivores. Conversely, if exposure occurs post-induction, grazer release could result in seaweed proliferation due to decreases in herbivore control. Herbivore fitness may also be impacted with the consumption of a relatively higher proportion of grazed tissue, perpetuating the influence of contaminants through multiple generations [48]. Understanding how these interactions are differentially impacted within the context of chemical contamination and other environmental stressors will offer us better insight and predictive capability on how these communities might shift in response to rapidly changing aquatic environments.
